# Calcium-Enriched Magnetic Core–Shell Mesoporous Nanoparticles for Potential Application in Bone Regeneration

**DOI:** 10.3390/nano15241904

**Published:** 2025-12-18

**Authors:** Despoina Kordonidou, Georgia K. Pouroutzidou, Nikoletta Florini, Ioannis Tsamesidis, Konstantina Kazeli, Dimitrios Gkiliopoulos, George Vourlias, Makis Angelakeris, Philomela Komninou, Panos Patsalas, Eleana Kontonasaki

**Affiliations:** 1 School of Physics, Aristotle University of Thessaloniki, 541 24 Thessaloniki, Greece; dkordoni@physics.auth.gr (D.K.); gpourout@physics.auth.gr (G.K.P.); nflori@physics.auth.gr (N.F.); kkazeli@physics.auth.gr (K.K.); gvourlia@auth.gr (G.V.); agelaker@auth.gr (M.A.); komnhnoy@auth.gr (P.K.); ppats@physics.auth.gr (P.P.); 2 Department of Prosthodontics, Faculty of Health Sciences, School of Dentistry, Aristotle University of Thessaloniki, 541 24 Thessaloniki, Greece; itsamesidis@auth.gr; 3 Laboratory of Chemical and Environmental Technology, Department of Chemistry, Aristotle University of Thessaloniki, 541 24 Thessaloniki, Greece; dgiliopo@chem.auth.gr

**Keywords:** Fe_3_O_4_ nanoparticles, mesoporous silica, calcium doping, magnetic core–shell nanoparticles, bone regeneration, biocompatibility, TEM, VSM

## Abstract

Magnetite (Fe_3_O_4_) nanoparticles are biocompatible, non-toxic, and easily functionalized. Coating them with mesoporous silica (mSiO_2_) offers high surface area, pore volume, and tunable surface chemistry for drug loading. In this study, Fe_3_O_4_ magnetic nanoparticles were synthesized and coated with mSiO_2_ shells enriched with calcium ions (Ca^2+^), aiming to enhance bioactivity for bone regeneration and tissue engineering. Different synthesis routes were tested to optimize shell formation Their characterization confirmed the presence of a crystalline Fe_3_O_4_ core with partial conversion to maghemite (Fe_2_O_3_) post-coating. The silica shell was mostly amorphous and the optimized samples exhibited mesoporous structure (type IVb). Calcium incorporation slightly altered the magnetic properties without significantly affecting core crystallinity or particle size (11.68–13.56 nm). VSM analysis displayed symmetric hysteresis loops and decreased saturation magnetization after coating and Ca^2+^ addition. TEM showed spherical morphology with some agglomeration. MTT assays confirmed overall non-toxicity, except for mild cytotoxicity at high concentrations in the Ca^2+^-enriched sample synthesized by a modified Stöber method. Their capacity to induce human periodontal ligament cell osteogenic differentiation, further supports the potential of Fe_3_O_4_/mSiO_2_/Ca^2+^ core–shell nanoparticles as promising candidates for bone-related biomedical applications due to their favorable magnetic, structural, and biological properties.

## 1. Introduction

Magnetic nanoparticles (MNPs) have attracted considerable attention in biomedical research due to their controllable size, magnetic responsiveness, and versatile surface functionalization [1]. These properties enable applications in drug delivery, imaging, hyperthermia, and tissue engineering [2,3,4,5]. However, bare MNPs often face challenges such as aggregation and structural instability under physiological conditions, which limit their practical use.

**Figure 1:**
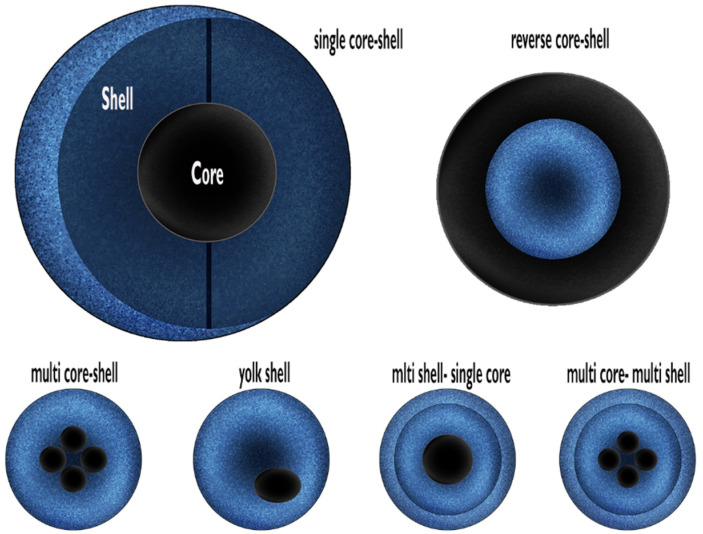
Classification of core–shell nanostructures. Redrawing from Singh R et al. [8].

To address these limitations, core–shell architectures, in which magnetic cores are coated with a protective shell such as mesoporous silica (mSiO_2_), have been developed (Figure 1). The shell not only preserves magnetic properties and enhances stability, but also provides a platform for surface functionalization and incorporation of therapeutic ions, opening avenues for multifunctional biomedical applications [6]. Core–shell nanoparticles are typically synthesized via bottom-up approaches, which allow precise control over particle size and uniform shell coating, and can be composed of various organic and inorganic materials [6,7]. The shell additionally protects the magnetic core from oxidation, improves biocompatibility, enables controlled circulation and drug release, and ensures optimal nanoparticle dispersion, making these systems suitable for advanced biomedical applications such as magnetic hyperthermia, bone regeneration, and drug delivery [7,8,9,10,11,12].

Mesoporous silica core–shell MNPs combine the advantages of high surface area and pore size of the mesoporous shell structure, and the biocompatibility of silica, with their magnetic properties from the core, leading to versatile nanoparticles. One profound benefit of these NPs is that the shell silica network can incorporate various therapeutic ions, such as zinc, copper, magnesium, etc., that can provide them with additional features and functionalities. For example, calcium is a fundamental component of bone mineral hydroxyapatite (Ca_10_(PO_4_)_6_(OH)_2_). By incorporating calcium ions into the mesoporous silica network, these NPs can release them in the surrounding environment upon degradation, promoting osteogenic differentiation, bone regeneration [13], and modulation of intracellular calcium levels, which are crucial in signaling pathways that drive these processes. Calcium ions can trigger the deposition of apatite (a component of bone) in physiological conditions, making these NPs suitable for use in bone repair and orthopedic applications. They can also enhance the adsorption of bioactive proteins or growth factors onto the surface of the shell, which can further stimulate cellular responses like osteogenesis or angiogenesis. In addition, they may enhance the biodegradability of the NPs, enabling faster and more robust calcium release into the surrounding tissues [13,14].

Although numerous Fe_3_O_4_/SiO_2_ core–shell magnetic nanoparticles have been explored for applications such as drug delivery and imaging, only a few studies have attempted to integrate magnetic targeting with ion-mediated biological activity in a single nanostructure [15,16]. Building upon an established mesoporous silica platform [8,14,15,16], we propose a multifunctional Fe_3_O_4_ mesoporous-SiO_2_ system enriched with Ca^2+^ ions. The design concept combines the high surface area, tunable porosity, and loading capacity of the mesoporous silica shell with the incorporation of calcium ions into the silica matrix to introduce additional osteogenic functionality through controlled ion release. As a key constituent involved in bone physiology, calcium plays a critical role in supporting osteogenic differentiation, guiding bone regeneration, and regulating intracellular signaling associated with these processes [12,13], while its incorporation into the mesoporous structure can enhance protein adsorption and overall bioactivity, thereby further contributing to regenerative outcomes [12,13,14].

The novelty of this study lies in uniting magnetic responsiveness, mesoporous-mediated therapeutic loading, and calcium-induced osteogenic potential within a single core–shell architecture, an approach that remains insufficiently addressed in the current literature. However, achieving a stable, bioactive Fe_3_O_4_/mSiO_2_/Ca^2+^ architecture requires careful optimization of synthesis routes to balance magnetic, structural, and biological performance. Therefore, this study aimed to synthesize and assess Ca^2+^-enriched Fe_3_O_4_/mSiO_2_ core–shell nanoparticles produced through different fabrication approaches. Comprehensive characterization, including structural, magnetic, morphological, cytocompatibility analyses and osteogenic differentiation, was conducted to determine how synthetic variations influence nanoparticle properties relevant to bone tissue engineering applications [8,12,13,14].

## 2. Materials and Methods

### 2.1. Chemicals

The materials used for the synthesis of magnetic Fe_3_O_4_ cores were ferric chloride hexahydrate (FeCl_3_•6H_2_O, 98%), ferrous chloride tetrahydrate (FeSO_4_•7H_2_O, 98%), and ammonium hydroxide (NH_4_OH, 25%). For the shell’s synthesis, tetraethyl orthosilicate (TEOS), cetyltrimethyl ammonium bromide (CTAB), ammonium hydroxide (25 wt%), ethanol (CH_3_CH_2_OH), calcium nitrate tetrahydrate (Ca(NO_3_)_2_•4H_2_O), triethanolamine (C_6_H_15_NO_3_), and chlorobenzene (C_6_H_5_Cl) are used across the different procedures. All reagents were from Merck, Darmstadt, Germany.

### 2.2. Synthesis of Fe_3_O_4_ (Core)

For the synthesis of the magnetic core, NPs of Fe_3_O_4_ were selected, which, together with Fe_2_O_3_, are biocompatible, and the chemical co-precipitation method was used [17]. FeCl_3_ (7.0003 g) and Fe_3_SO_4_ (3.6000 g) were dissolved in 50 mL of double-distilled water (ddH_2_O). After dissolution, the two aqueous solutions were mixed within a three-necked flask, and the sample was heated for 15 min at 60 °C under a flow of inert argon gas (Ar) and a magnetic stirring speed of 450 rpm. pH measurements, up to this stage, showed a value of pH_before_ = 1.6. Subsequently, a gradual (drop-by-drop) addition of ammonia (NH_3_) (50 mL) solution followed, which raised the pH value to 10. The solution was subsequently subjected to 90 min of stirring and parallel magnetic stirring at 450 rpm and 60 °C, to enhance nucleation and achieve a narrower size distribution. During nucleation, Ar flow was controlled through viscous glycerol. At the end of the nucleation (deep black-colored solution), the stirring and heating stopped, and the magnetic rod was removed from the flask [17]. The black solution was placed over a strong permanent magnet (NdFeB) to rapidly and effectively separate the precipitate from the solution, completing the submersion process. The following chemical equation presents the possible reaction for the Fe_3_O_4_ NPs’ formation [17]:

2FeCl_3_⋅6H_2_O + FeSO_4_⋅7H_2_O + 8NH_4_OH → Fe_3_O_4_ + 6NH_4_Cl + (NH_4_)_2_SO_4_ + 23H_2_O, (1)

Τhe sediment was subjected to ten washes with ddH_2_O, while re-using the same strong magnet, until the strong ammonia odor disappeared. Measurements of pH during the 4th and 10th washing showed values of 9.7 and 8.91, respectively. The repeated washes were performed to reduce the amount of salt coming from other reactions that probably took place in the solution [17]. The washed solution was centrifuged, transferred to Falcon tubes, and dried overnight in ceramic containers in an oven at 70–80 °C The dried material was finally ground using a mortar and pestle to produce fine Fe_3_O_4_ powder.

### 2.3. Synthesis of mSiO_2_ (Shell Composition)

**Figure 2:**
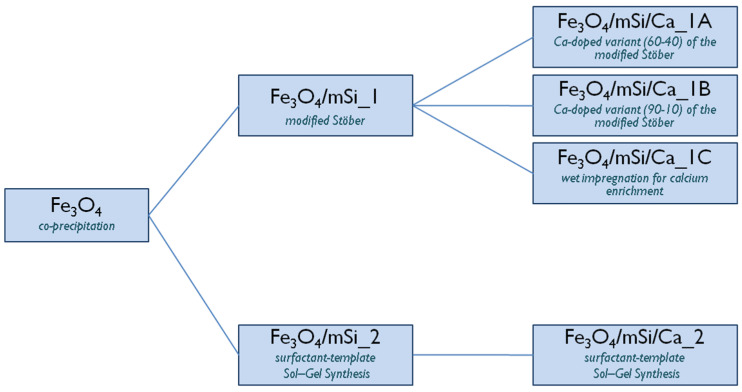
Classification of the different shell and Ca-enriched shell synthesis methods.

Different experimental procedures were followed to synthesize the shell. In the first approach, a modified Stöber method was used (sample Fe_3_O_4_/mSi_1_), while the second approach employed a surfactant-templated sol–gel synthesis (sample Fe_3_O_4_/mSi_2_). For calcium enrichment, a calcium-doped variant of the modified Stöber synthesis was performed with different TEOS:Ca ratios (60:40 and 90:10) for samples Fe_3_O_4_/mSi/Ca_1A and Fe_3_O_4_/mSi/Ca_1B, respectively. Sample Fe_3_O_4_/mSi/Ca_1C was prepared via a wet impregnation method, while the synthesis of sample Fe_3_O_4_/mSi/Ca_2 followed a calcium-doped variant of the surfactant-templated sol–gel procedure (Figure 2).

#### 2.3.1. Experimental Procedure 1

##### Sample Fe_3_O_4_/mSi_1 (Modified Stöber)

In the first case, for the synthesis of mSiO_2_/Fe_3_O_4_, the modified Stöber method was used [18]. More specifically, 0.1 g Fe_3_O_4_ NPs previously synthesized were added to 80 mL ddH_2_O, and the mixture was subjected to dispersion at 70 °C using an ultrasonic homogenizer for a duration of 10 min (10″–30″ pulse″ on/off, respectively). In the second stage, 60 mL of ethanol and 0.3 g CTAB were added, the solution was then subjected to manual stirring for 15 min, and then the ultrasonic homogenizer was used once more (10″–30″ pulse on/off). CTAB is a quaternary ammonium salt that acts as a surfactant. Then 1.12 mL, 25%wt NH_3_ was added into the solution under ultrasonic homogenization (10″–30″ pulse on/off). Afterwards, 0.3 mL of the silica source, TEOS was added (drop-by-drop), while the sample was subjected to mechanical, manual stirring for 15′. The final solution was placed in a shaker for 6 h at 37 °C and 250 rpm [14].

At the end of the 6 h reaction, the product was separated from the suspension by a NdFeB magnet (external magnetic field). Next, two washing steps were performed to remove nitrates, the first with ethanol and the second with ddH_2_O, each for 5 min at 8000 rpm. The sample was then dried overnight at 60 °C. The next day, the sample was placed in a flask and subjected to minimal pressure to relieve any residual stress, resulting in a fine powder. Finally, the material underwent heat treatment (calcination) at 350 °C for 2 h to remove the CTAB.

##### Sample Fe_3_O_4_/mSi/Ca_1A (Ca-Doped Variant 60:40 of the Modified Stöber)

For the enrichment of the silica shell with calcium ions, the same synthesis procedure was followed until the dropwise addition of TEOS. After 30′ of shaking, at 37 °C and 250 rpm, 0.157 g of hydrated Ca nitrate Ca(NO_3_)_2_•4H_2_O was added to the solution, in a chemical composition of TEOS:Ca^2+^ in a 60:40 ratios, respectively. The new solution was dispersed for 5′ (10″–30″ pulse on/off), stirred once again for another 5′30 h at 37 °C and 250 rpm to ensure complete reaction. After stirring, the product was separated from the suspension through a strong magnet. Two washing procedures were performed, followed by drying and calcination as described above.

##### Sample Fe_3_O_4_/mSi/Ca_1B (Ca-Doped Variant 90:10 of the Modified Stöber)

To explore the amount of TEOS on the properties of the shell, the same shell synthesis procedure was followed once more with a chemical composition of TEOS:Ca^2+^ in a 90:10 ratio. More specifically, the previously described procedure of sample Fe_3_O_4_/mSi_1 was followed up to the point of TEOS addition. Following 30 min of shaking at 37 °C and 250 rpm, 0.027 g of Ca(NO_3_)_2_•4H_2_O was introduced into the solution, maintaining a TEOS:Ca^2+^ molar ratio of 90:10. The resulting mixture was subjected to ultrasonic dispersion for 5 min (10″–30″ pulse on/off), followed by an additional stirring phase lasting 5′30 h under the same temperature and agitation conditions, to ensure complete reaction. Upon completion, the product was magnetically separated from the suspension. Subsequent washing steps with ethanol and deionized water were performed, and the sample was then dried and calcined as previously described.

##### Fe_3_O_4_/mSi/Ca_1C (Wet Impregnation for Calcium Enrichment)

Another experimental procedure for the Ca^2+^ enrichment was performed with the method of wet-impregnation [15], to receive a mesoporous silica and a Ca-doped shell. First, Fe_3_O_4_/mSi_1 sample (0.10 g) was dispersed in ddH_2_O (10.0 mL) via ultrasonic treatment. Then, Ca(NO_3_)_2_•4H_2_O (30 mg) was added to the mixture, and the resulting solution was evaporated at 80 °C and subsequently calcined at 600 °C for 3 h to convert Ca(NO_3_)_2_ into CaO.

#### 2.3.2. Experimental Procedure 2

##### Sample Fe_3_O_4_/mSi_2 (Surfactant-Templated Sol–Gel Synthesis)

For this approach, 300 mg Fe_3_O_4_ NPs were mixed with 0.05 g triethanolamine and 30 mL of ddH_2_O [16]. This first solution was dispersed in an ultrasonic homogenizer for 15’ (10″–30″ pulse on/off). A second solution of 1.5 g CTAB and 15 mL ddH_2_O was magnetically stirred for 15’ at 60 °C, and then the two solutions were mixed and left to react for 1 h at 60 °C [16]. Subsequently, 14.5 mL of chlorobenzene (CB) and 1.5 mL of TEOS were added to the final solution, which was then dispersed using an ultrasonic homogenizer for 5 min (10–30 s pulse on/off). The mixture was afterwards stirred in a shaker at 37 °C and 100 rpm for 15 h. Once stirring was complete, the sample was washed twice with ethanol and dried overnight in an oven at 60 °C. The next day, the solution was calcinated for 2 h at 350 °C similar to synthesis procedure 1 [16].

##### Sample Fe_3_O_4_/mSi/Ca_2 (Ca-Doped Variant of the Surfactant-Templated Sol–Gel Synthesis)

Enrichment of the shell with Ca ions was also performed in the second synthesis route. The procedure followed the same steps described in the previous section for sample Fe_3_O_4_/mSi_2_, up to the point at which 1.5 mL of TEOS was added to the solution. After that, the solution was dispersed in an ultrasonic homogenizer for 5’ (10″–30″ pulse on/off) and then placed in a shaker for 30’ at 37 °C, 100 rpm. After half an hour of shaking, 0.785 g of Ca(NO_3_)_2_•4H_2_O was added to the solution which was placed once more into the shaker to complete 15 h of shaking [16]. After shaking, the sample was washed with ethanol twice, dried overnight at 60 °C and the next day calcined for 2 h at 350 °C [16].

Sample names and composition for all experimental procedures are presented in Table 1.

### 2.4. Materials Characterization

X-ray diffraction (XRD) patterns of the materials were recorded using a Rigaku Ultima type diffractometer (Rigaku Corporation, Tokyo, Japan), with a Cu tube mounted on it and a Ni filter for CuKa radiation (*λ* = 0.1542 Å). The scanning angle range 2θ was between 5 and 90°, with a step size of 0.05°, and a scanning speed of 0.05° 2θ/s. The Jade software version 6.0.3 was used for identification.

X-ray photoelectron spectroscopy (XPS) analysis was performed for Fe_3_O_4_/mSi_1 and Fe_3_O_4_/mSi/Ca_1C samples on a Kratos Analytical AXIS Ultra DLD system (Kratos Analytical, Manchester, UK) with an Al Kα X-ray source (hν = 1486.6 eV), under ultra-high vacuum conditions (~10^−9^ torr). By applying 105 W (7 mA/12 kV) to the X-ray source, the analyzer with a pass energy of 160 eV recorded wide-scan (survey) spectra. High-Resolution (HR) regions were recorded with a pass energy of 20 eV, applying 120 W (10 mA/15 kV) during a three-sweep scan, except for the Ca 2p regions, where a five-sweep scan was used for better signal-to-noise ratio. The C 1s peak at 284.6 ± 0.2 eV, corresponding to C–C bonds from environmental contamination, was used for the spectra calibration.

For quantitative analysis, Shirley (non-linear) baselines were used to subtract the background from the HR peaks, and the atomic percentages were calculated using the RSF factors of each element. Gaussian (70%) and Lorentzian (30%) components, as well as asymmetric curves (depending on the chemical state of each element), were used to fit the experimental curves for the chemical state analysis.

Fourier-transform infrared (FTIR) measurements were carried out using potassium bromide (KBr) pellets the background. The synthesized MNPs powder was mixed with KBr powder at a 1:100 ratio. Next, the samples were compressed in a hydraulic press under 7 tons of pressure to produce 13 mm diameter pellets. Transmittance spectra were obtained with a PerkinElmer FTIR Spectrum 1000 spectrometer, in the mid-infrared (MRI) range (4000–400 cm^−1^), with a resolution of 4 cm^−1^ and 32 scans.

The magnetic properties of the nanoparticles were characterized using a 1.2H/CF/HT Oxford Instruments Vibrating Sample Magnetometer (VSM). A magnetic sample is deposited at the center of both four small collection coils (contributing to the inductive signal) and at the center of two electromagnet poles that produce a highly homogeneous magnetic field. By means of a sinusoidal signal, emitted by the oscillator, a vertical signal—vibration—is identified by means of a transducer. The sample maintains a frequency of 60 Hz and an amplitude of 1.5 mm. The external field used remains constant for the purpose of magnetizing the sample only, thus not affecting the voltage. The maximum external magnetic field amplitude used in this VSM device is 1.2 T. The measured samples are in powder form and weigh at least 0.5 mg, to achieve the minimum required signal while reducing the signal noise [19,20]. Finally, the hysteresis loops for each type of MNPs were recorded at room temperature.

For Transmission Electron Microscopy (TEM) analysis, a small amount of each MNP sample was dispersed in ethanol and sonicated for 30 min. Drops of the ethanol-MNP samples were dispersed on a 300-mesh Cu carbon-coated grid and dried at room temperature. The TEM instrument (JEOL JEM Cold Field Emission Gun (CFEG) TEM/STEM F200, JEOL Ltd., Tokyo, Janpan) was operated at 200 kV, with 0.19 nm point-to-point resolution, equipped with a RIO GATAN CMOS 9 Mpixel bottom-mounted camera (Gatan, Inc., San Diego, CA, USA). For Fe_3_O_4_/mSi/Ca_1A, Fe_3_O_4_/mSi/Ca_1B, and Fe_3_O_4_/mSi/Ca_2 samples, an additional EDX detector (T-max 65, Oxford Instruments, Oxfordshire, UK) was employed. The analysis focused on morphology, particle size, and structure. In the case of Fe_3_O_4_/mSi/Ca_2 solution, the chemical composition of the NPs was also investigated.

TEM was employed to determine nanoparticle size statistics for more accurate average size measurements. TEM images from different regions of each MNP sample were analyzed, and the diameters of at least 300 nanoparticles per sample were measured using ImageJ version 1.8.0_172 (64-bit). The resulting size distributions were visualized as histograms in OriginLab, OriginPro 9.0 software. Smoothing of the average particle size data was performed to produce a histogram in normal or log-normal format. In the case of lognormal, the average particle size of each sample is calculated by fitting the particle size distribution histogram to the log-normal distribution function expressed by the relation:

(2) $$f\left(D\right)=\left(\frac{1}{\sqrt{2\pi }\sigma_{D}}\right)exp\left[-\frac{{ln}^{2}\left(\frac{D}{D_{0}}\right)}{2\sigma^{2}}\right],$$

D is the average particle size, $\sigma_{D}$ is the standard deviation [19]. From each histogram and by Origin’s library, the values of Inmean and InSd were obtained and the values of $D_{0}$ and $\sigma_{Sd}$ were calculated as:

(3) $$D_{0}=e^{Inmean},\;\sigma_{Sd}=e^{InSd},\;e\approx 2.718,$$

Subsequently, according to the sample measurement, a series of HRTEM images was obtained, equivalent to that of the PSA300 system’s software. Particles were separated from the background by setting the threshold function parameter [19,20].

### 2.5. Biological Characterization

#### 2.5.1. Cytotoxicity

To investigate MNPs cytotoxicity, the indirect MTT (3-(4,5-dimethylthiazol-2-yl)-2,5-diphenyltetrazolium bromide) assay was employed. Eluates were collected after 24 h incubation time with the NPs at different concentrations (C1 = 0.5 mg/mL and C2 = 0.25 mg/mL) in Dulbecco’s minimal essential medium, DMEM. In detail, eluates were performed in sterile chemically inert closed containers using aseptic techniques, and passed through 0.22 µm filters. More specifically, human gingival fibroblasts (HGFs) (1 × 10^3^ cells per well) were seeded in 96-well plates to allow cell attachment. After 24 h of cell culture, DMEM was removed and replaced with eluates of the three tested materials. HGFs cultured in DMEM supplemented with FBS and antibiotics served as the positive control. Analysis of mitochondrial activity and thus cell proliferation was succeeded by measuring the mitochondrial dehydrogenase activity of metabolically active cells, verified by the capability of transforming the yellow tetrazolium salt into blue formazan crystals [20,21]. MTT solution was added in each well (10% of the total volume per well) and 3 h incubation at 37 °C and 5% CO_2_ followed. After this period, the medium containing the MTT solution was discarded, and the insoluble formazan was dissolved in dimethyl sulfoxide (DMSO) [21,22]. Cell viability was evaluated by measuring the optical density with an ELISA-spectrophotometer (Epock, Biotek, Winooski, VT, USA) at a double wavelength (570–630 nm). The experiments were performed in triplicate, and the results are presented as a % compared to the positive control.

#### 2.5.2. Reactive Oxygen Species (ROS) Levels

Human periodontal ligament cells (hPDLCs) were seeded at a density of 4 × 10^4^ cells/well in 48-well plates and allowed to attach for 24 h before experimentation. Reactive Oxygen Species (ROS) levels were assessed on days 1 and 3 following exposure to MNPs. After incubation with the NPs (0.25 μg/mL) for 1 and 3 days, consequently, cells were washed with PBS and incubated with DCFH-DA solution (according to manufacturer’s instructions) for 30 min at 37 °C. Fluorescence intensity (Ex/Em: 485/535 nm) was measured using a Tecan fluorometer with black 96-well microplates to quantify ROS production; their background signals were subtracted from the respective readings. Maximal emission corresponded to the fluorescence measured in a reaction containing only H2DCFDA and H_2_O_2_ at a final concentration of 3 mM, as previously performed. This concentration was chosen based on a standard curve generated with H_2_O_2_ concentrations ranging from 0.1 to 5 mM, which allowed fluorescence to be recorded without exceeding the detection limit. A final concentration of 3 mM H_2_O_2_ produced the highest fluorescence values without signal overflow.

#### 2.5.3. Osteogenic Differentiation

Human periodontal ligament cells (hPDLCs) were seeded at a density of 4 × 10^4^ cells/well in 48-well plates and allowed to attach for 24 h before experimentation. Fe_3_O_4_/mSi, Fe_3_O_4_/mSi/Ca_1A, Fe_3_O_4_/mSi/Ca_1B, and Fe_3_O_4_/mSi/Ca_1C MNPs were pre-treated for 1 day in DMEM under sterile conditions prior to cell exposure. MNPs were used at a final concentration of 0.25 μg/mL, and all conditions were performed in triplicate. Osteogenic differentiation was induced using an osteogenic medium (OM), prepared according to a previously described protocol [23]. The following experimental groups were established:

(1) hPDLCs cultured with each MNP type in OM;
(2) hPDLCs cultured in OM without NPs (positive control).

For all conditions, the culture medium was refreshed every 2 days. The endpoints for each assay were as follows: ALP activity (day 7 and day 14), and ARS staining (day 21).

#### 2.5.4. Alkaline Phosphatase Activity

Alkaline Phosphatase (ALP) activity was analyzed on day 7 and day 14. After incubation with culture medium in the presence or absence of NPs (0.25 μg/mL), both cell lysates and culture supernatants were collected. Samples were mixed with an ALP reaction buffer (Apollo Scientific, BI4545/BIN0446, Manchester, UK), following the procedure described previously [23,24].

The enzymatic conversion of p-nitrophenyl phosphate (pNPP) was quantified by measuring the absorbance at 405 nm. ALP activity values were compared with those obtained from hPDLCs cultured without NPs under identical medium conditions.

#### 2.5.5. Alizarine Red Staining

Calcium mineral deposition was evaluated on day 21 via Alizarin Red S (ARS) Staining. hPDLCs were seeded in 12-well plates at 4 × 10^4^ cells/well and cultured with or without the MNPs formulations (0.25 μg/mL) in OM. Media were replaced every 2 days. Parallel wells containing MNPs without cells were included to enable subtraction of OD values originating from NPs. At the endpoint, cultures were fixed and stained with Alizarin Red S (Sigma-Aldrich, St. Louis, MO, USA). Mineralized nodules were visualized using an inverted microscope. For quantification, the bound dye was eluted using 10% (*w*/*v*) cetylpyridinium chloride for 20 min at room temperature, and absorbance was measured at 540 nm using a microplate spectrophotometer.

## 3. Results

### 3.1. XRD

**Figure 3:**
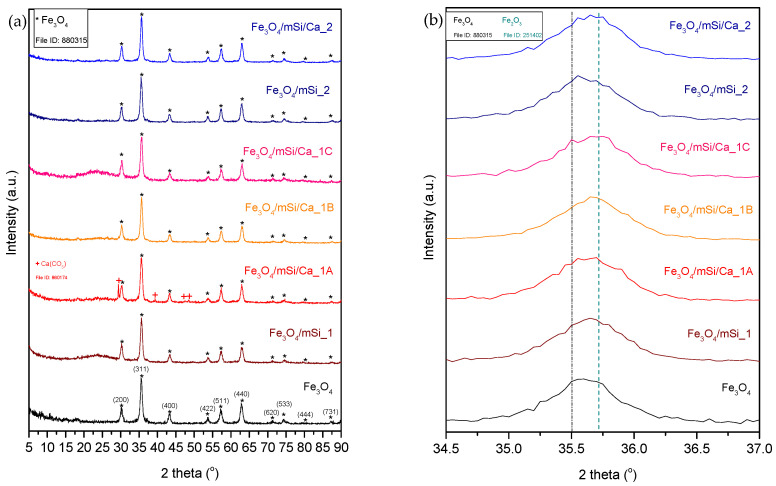
(**a**) X-ray diffraction peak plots for all magnetic core–shell NPs synthesized. (**b**) Enlarged region between 34 and 37° 2θ. * = diffraction peak corresponding to Fe_3_O_4_, + = diffraction peak corresponding to Ca(CO_3_).

Figure 3 shows the XRD patterns of Fe_3_O_4_, Fe_3_O_4_/mSi_1, Fe_3_O_4_/mSi/Ca_1A, Fe_3_O_4_/mSi/Ca_1B, Fe_3_O_4_/mSi/Ca_1C, and Fe_3_O_4_/mSi_2, Fe_3_O_4_/mSi/Ca_2 samples. Fe_3_O_4_ NPs exhibit diffraction peaks localized at specific angles 2θ and corresponding to crystal surfaces of magnetite (Fe_3_O_4_) diffraction peaks (Jade tab: #880315). The observed 2θ angles included 30°, 35.5°, 43.3°, 53.8°, 57.2°, 62.8°, 71.2°, 74.3°, 79.65°, and 87.2°, which map respectively to the (220), (311), (400), (422), (511), (440), (620), (533), (444), and (731) crystalline surfaces of magnetite [16,17].

All diffraction peaks correspond to the characteristic face-centered cubic (fcc) structure, although secondary phases (peaks at 2θ ≥ 71.2°) are also observed. The crystal structure and high-intensity main diffraction peaks of the samples reveal high crystallization for the magnetite-rich sample, while the wide reflections of the sample highlight its nanocrystalline nature [6]. At 35.7 2θ, there is a tendency for a secondary peak attributed to Fe_2_O_3_. All Fe_3_O_4_ peaks are still distinct after coating the magnetic core with a SiO_2_ shell and after shell’s enrichment with Ca^2+^ in all samples. This indicates the good preservation of the magnetic core. After coating and shell-enrichment, samples do not undergo a significant change in crystallinity compared to the control (Fe_3_O_4_), except for a faint decrease in diffraction peaks’ intensity due to the addition of an amorphous shell. At the same time, during shell formation, the secondary peak of Fe_2_O_3_ is distinctly visible in the diffraction patterns. No new peaks corresponding to a calcium crystal phase were observed, with the notable exception of a strong double peak in the Fe_3_O_4_/mSi/Ca_1A sample at 2θ = 29.35° and 30.22°, along with weaker peaks at 39.35°, 47.5°, and 48.4°. This indicates that calcium ions are well dispersed within the silica shell [16,17].

### 3.2. XPS

**Figure 4:**
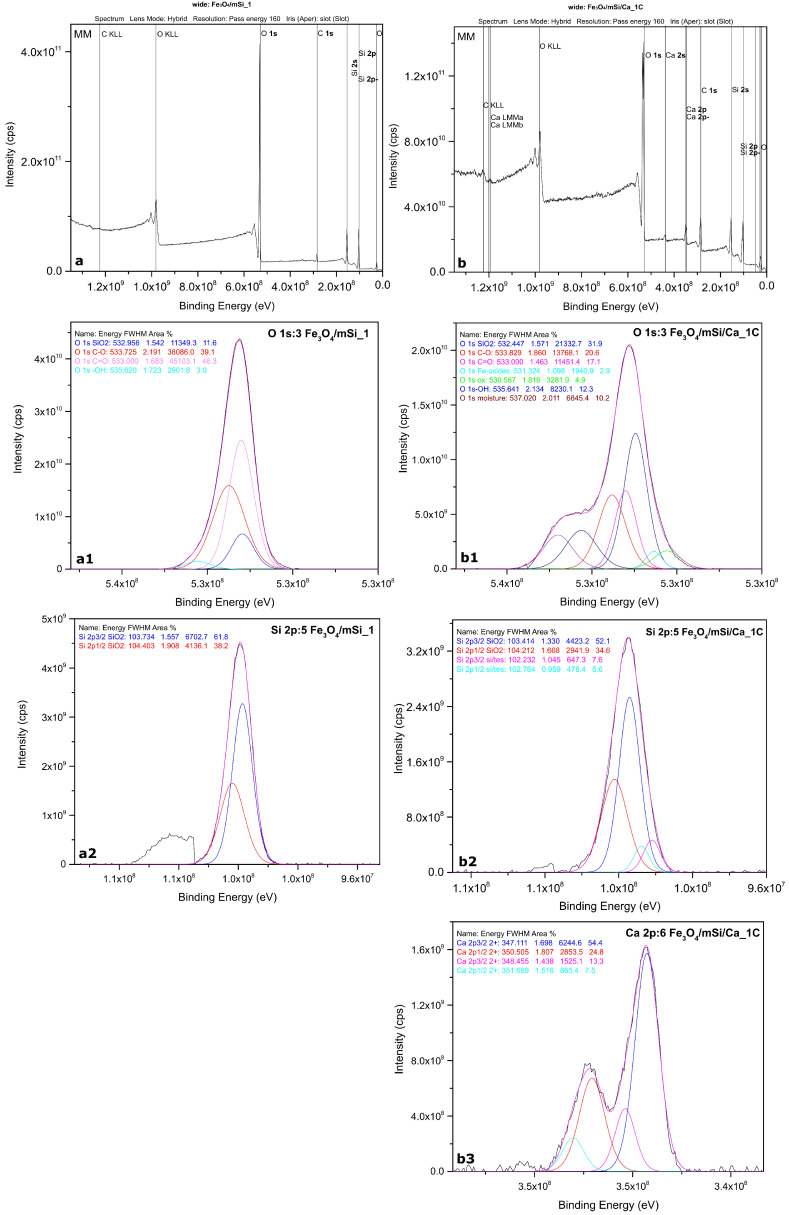
XPS spectrums: (**a**) wide scan of Fe_3_O_4_/mSi_1 MNPs, (**b**) wide scan of Fe_3_O_4_/mSi/Ca_1C MNPs, (**a1**) O of Fe_3_O_4_/mSi_1 MNPs, (**a2**) Si of Fe_3_O_4_/mSi_1 MNPs, (**b1**) O of Fe_3_O_4_/mSi/Ca_1C MNPs, (**b2**) Si of Fe_3_O_4_/mSi/Ca_1C MNPs and (**b3**) Ca of Fe_3_O_4_/mSi/Ca_1C MNPs.

The recording of the wide/survey spectrum for the Fe_3_O_4_/mSi_1 sample yielded peaks corresponding only to O and Si. Si at 0 nm is in an oxidative state (IV), in the form of SiO_2_ (Figure 4).

Regarding Fe_3_O_4_/mSi/Ca_1C wide-scan spectra yielded an almost identical stoichiometry, with the exception that additional Ca peaks appear in the present sample, confirming its incorporation (Figure 4). The behavior of Si is similar to that of the previous sample and Ca is present in the Ca(II) oxidation state. The fitted Ca 2p spectra are presented below. The quantification results of both samples are presented in Table 2.

### 3.3. FTIR

**Figure 5:**
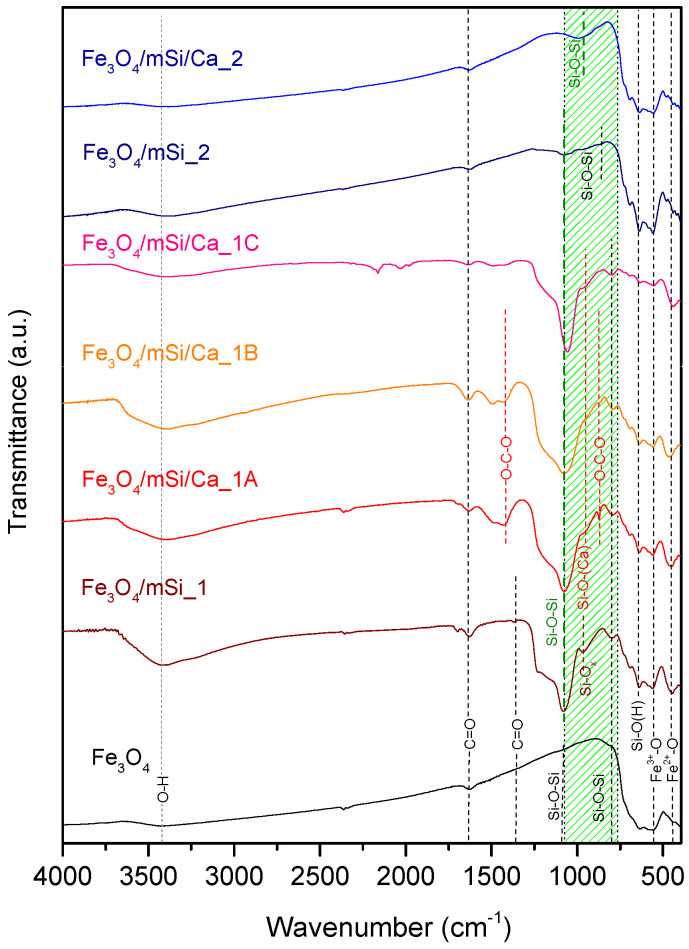
FTIR spectra for Fe_3_O_4_, Fe_3_O_4_/mSi_1, Fe_3_O_4_/mSi/Ca_1A, Fe_3_O_4_/mSi/Ca_1B, Fe_3_O_4_/mSi/Ca_1C, Fe_3_O_4_/mSi_2 and Fe_3_O_4_/mSi/Ca_2 MNPs. The green highlighted area corresponds to Si–O and Si–O–X bonds.

Figure 5 shows the FTIR spectra in Transmittance mode of all tof the synthesized samples. For the Fe_3_O_4_ sample, transmittance bands appear at 562 cm^−1^ and, more weakly, at 440 cm^−1^, attributed to stretching vibrations of Fe–O bonds [16,17]. The band of 562 cm^−1^ corresponds to Fe^3+^–O at tetrahedral sites, while the band at 440 cm^−1^ corresponds to Fe^2+^–O vibrations at octahedral sites [25,26]. Weak bands appear at 818 cm^−1^ and 1104 cm^−1^, which are attributed to antisymmetric and symmetric stretching vibrations of Si–O–Si bonds, respectively [15,17]. Subsequently, the presence of carboxylic groups (C=O) is observed at 1624 cm^−1^ and weakly at 1364 cm^−1^. Finally, the peak at 3418 cm^−1^, corresponds to O–H stretching vibrations, due to the interaction of water (H_2_O) molecules with the surface of the MNPs. The presence of hydroxyl groups ion Fe_3_O_4_ NPs facilitates modification through the incorporation of additional materials [15,16,17,25,26].

Fe_3_O_4_/mSi_1 sample’s peak at 446 cm^−1^ (vibration between Fe^2+^–O bonds) is more pronounced after the silica coating, so it is likely that during shell coating, part of Fe_3_O_4_ was transformed into Fe_2_O_3_. A band in the 620–740 cm^−1^ region supports the presence of Fe_2_O_3_ phases [3,4]. The peak at 638 cm^−1^ refers to the symmetric stretching vibration between Si–O(H) bonds [13,15,16]. Peak at 968 cm^−1^ is attributed to asymmetric vibrations of Si–OH or SiO_x_ bonds due to the formation of an open silicate network [27,28]. The characteristic peaks at 789 cm^−1^ and 1082 cm^−1^ assigned to symmetric and antisymmetric Si–O–Si stretching [14,15], confirm successful silica coating. Similarly, in the spectra of Fe_3_O_4_/mSi/Ca_1_A, Fe_3_O_4_/mSi/Ca_1_B, and Fe_3_O_4_/mSi/Ca_1_C, the peak near 440 cm^−1^ appears more pronounced, indicating an increased formation of Fe_2_O_3_ following Ca enrichment [3,4]. The open silica network formation at ~968 cm^−1^ decreases in intensity, consistent with Si–O–Ca bond formation. Confirming XRD’s results, additional peaks appear at 1425 cm^−1^ and 874 cm^−1^ due to out-of-plane bending/asymmetric stretching of O–C–O bonds, respectively [29,30].

The analysis of the Fe_3_O_4_/mSi_2_ sample was consistent with the core’s characteristic peaks due to stretching vibrations primarily reflecting Fe^3+^–O stretching vibrations and to a lesser extent Fe^2+^–O vibrations. A weaker band in the 620–740 cm^−1^ region again suggests minor Fe_2_O_3_ formation. A peak at 638 cm^−1^ corresponds to Si–O(H) stretching, while bands at 876 cm^−1^ (antisymmetric Si–O–Si) and 1076 cm^−1^ (symmetric Si–O–Si) indicate the presence of silicates, but with lower intensity compared to the first synthesis. After Ca^2+^ enrichment (sample Fe_3_O_4_/mSi/Ca_2), the silicate contribution is limited to a shifted band at 994 cm^−1^ (symmetric Si–O–Si).

### 3.4. VSM

**Figure 6:**
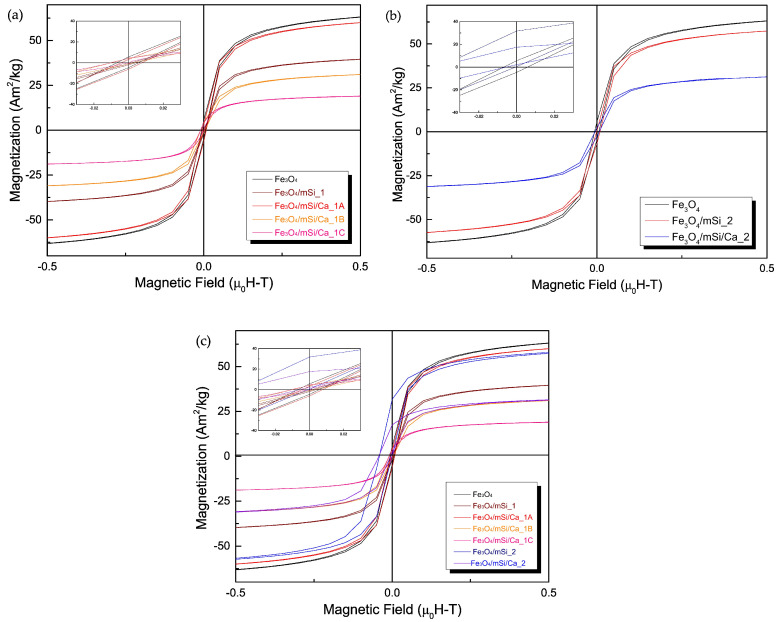
Hysteresis loops of (**a**) Fe_3_O_4_, Fe_3_O_4_/mSi_1, Fe_3_O_4_/mSi/Ca_1, Fe_3_O_4_/mSi/Ca_1A and Fe_3_O_4_/mSi/Ca NPs, (**b**) Fe_3_O_4_, Fe_3_O_4_/mSi_2 and Fe_3_O_4_/mSi/Ca_2 NPs and (**c**) all synthesized samples.

Figure 6a–c presents the hysteresis loops of samples synthesized via the first (Figure 6a) and second (Figure 6b) methods. All NPs, after coating, display hysteresis loops characteristic of Fe_3_O_4_ NPs, regardless of the synthesis route, but with notably reduced magnetization. In the first synthesis procedure, a slight decrease in magnetization is observed following coating, followed by an increase in the sample Fe_3_O_4_/mSi/Ca_1A with a TEOS:Ca^2+^ ratio of 60:40. This trend contrasts with the magnetization decrease noted in samples Fe_3_O_4_/mSi/Ca_1B and Fe_3_O_4_/mSi/Ca_1C, prepared with a TEOS:Ca^2+^ ratio of 90:10 (Figure 6a). For samples synthesized with the second method (Fe_3_O_4_/mSi_2 and Fe_3_O_4_/mSi/Ca_2, Figure 6b), magnetization decreases progressively after shell addition and calcium enrichment.

These results indicate that both the synthesis method and TEOS:Ca^2+^ ratio influence the magnetic properties, potentially due to variations in shell thickness and calcium incorporation efficiency. Saturation magnetization (M_S_) values measured under an external magnetic field of 0.5 [*μ*_0_H–T] are summarized in Table 3.

### 3.5. TEM

#### 3.5.1. Morphology, Structure and Composition of NPs

**Figure 7:**
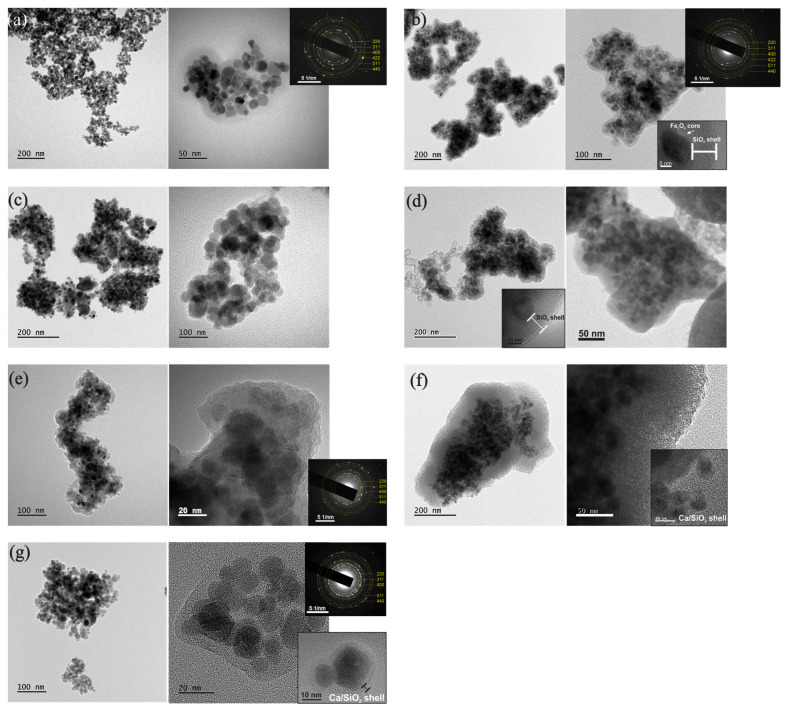
TEM1: Bright-field TEM images of (**a**) Fe_3_O_4_ NPs and corresponding SAED ring pattern of magnetite cubic Fe_3_O_4_ structure, (**b**) Fe_3_O_4_/mSi_1 NPs and corresponding SAED ring pattern of magnetite cubic Fe_3_O_4_ structure. The amorphous shell around the NP is shown as an inset, (**c**) Fe_3_O_4_/mSi_2 NPs, (**d**) Fe_3_O_4_/mSi/Ca_1A NPs and the existence of a Ca/SiO_2_ shell as an inset, (**e**) Fe_3_O_4_/mSi/Ca_1B NPs and the corresponding SAED ring pattern, (**f**) Fe_3_O_4_/mSi/Ca_1C NPs and the existence of thick Ca/SiO_2_ shell and (**g**) Fe_3_O_4_/mSi/Ca_2 NPs and corresponding SAED ring pattern along with the existence of Ca/SiO_2_ shell around the NPs.

**Figure 8:**
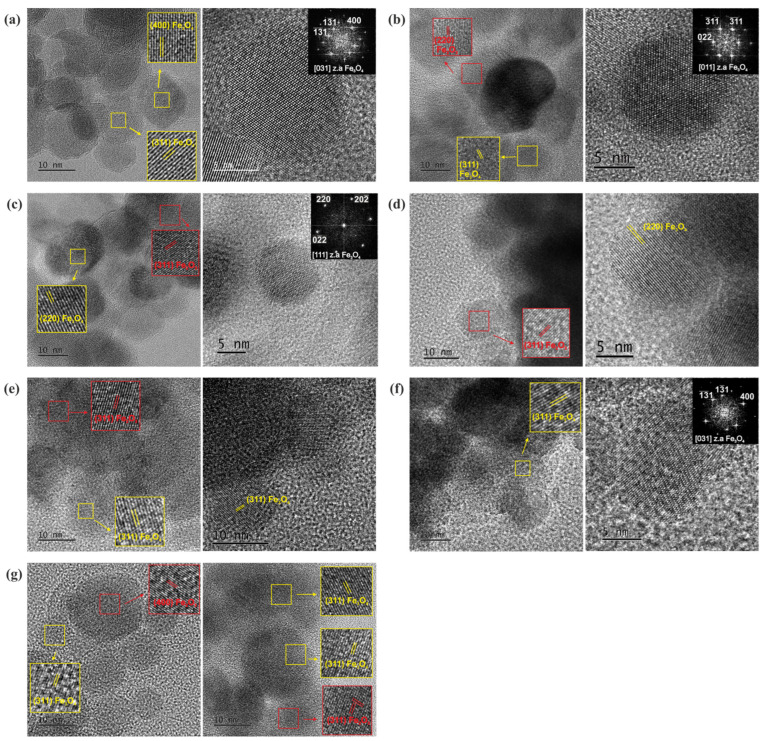
TEM 2: HRTEM images of (**a**) nearly spherical Fe_3_O_4_ NPs with excellent crystallinity and corresponding FTT pattern, (**b**) Fe_3_O_4_/mSi_1 NPs and a representative NP with corresponding FTT confirming the fcc structure, (**c**) Fe_3_O_4_/mSi_2 NPs and a representative NP with corresponding FFT confirming the atomic structure of Fe_3_O_4_ NPs projected along the <111> zone axis, (**d**) Fe_3_O_4_/mSi/Ca_1A, (**e**) Fe_3_O_4_/mSi/Ca_1B, (**f**) Fe_3_O_4_/mSi/Ca_1C and (**g**) Fe_3_O_4_/mSi/Ca_2 NPs. The detected lattice fringes of (220), (311), and (400), highlighted in yellow rectangles, correspond to the cubic structure of magnetite (Fe_3_O_4_), while lattice fringes highlighted in red rectangles correspond to the cubic structure of maghemite (Fe_2_O_3_), indicating phase coexistence.

The microstructure of the Fe_3_O_4_ NPs, in both procedures 1 and 2, was investigated using TEM, SAED, and HRTEM imaging modes. From the bright-field TEM images (Figure 7), two types of shapes were observed in terms of Fe_3_O_4_ NPs’ morphology: the spherical (smaller NPs) with diameters up to 13 nm, and a nearly rectangular shape for larger NPs. The average size of spherical NPs was calculated to be close to 11.7 nm. SAED analysis revealed that the NPs exhibit the characteristic ring pattern of Fe_3_O_4_’s crystal structure, as shown in the insets of Figure 8. HRTEM images (Figure 8) showed an almost spherical shape of the NPs with excellent crystallinity, while the spatial frequencies visible through FFT confirmed the face-centered cubic (fcc) structure of Fe_3_O_4_. The shape, size, and crystallinity degree of the NPs did not show significant changes after coating in either of the two synthesis procedures. The particles exhibited a small size distribution, were aggregated, and were overly surrounded by a successfully formed amorphous SiO_2_ shell, which increased the Fe_3_O_4_ particles’ average diameter.

**Figure 9:**
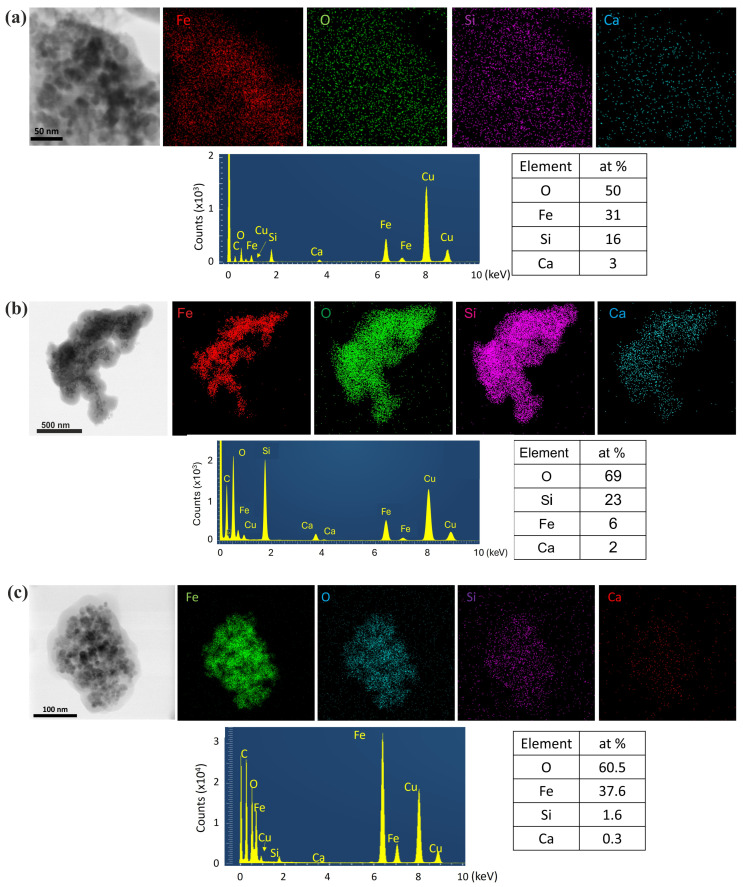
TEM 3: STEM-EDX elemental mapping and corresponding EDX spectra for (**a**) Fe_3_O_4_/mSi/Ca_1A sample, (**b**) Fe_3_O_4_/mSi/Ca_1C, and (**c**) Fe_3_O_4_/mSi/Ca_2 samples, showing the distribution and atomic content of all detected elements. The additional C and Cu peaks originate from the carbon-coated Cu TEM grid.

In particular, synthesis procedure 1 revealed a shell thickness of ~10 nm (Figure 7b). The NPs exhibited darker contrast compared to the brighter SiO_2_ shell, while the formed pores contributed to future dispersion of alkaline species [15,16]. The corresponding SAED ring patterns, shown as insets in Figure 7b,e, depict the Fe_3_O_4_ crystal structure. The sequence of the diffraction rings, from the inner to the outer, corresponds to the interplanar spacing of {220}, {311}, {400}, {422}, {511}, and {440} lattice planes of the Fe_3_O_4_ magnetite structure (cubic F$\bar{d}$3m). The HRTEM images in Figure 8b,d–f reveal the cubic structure of Fe_3_O_4_ along with a minority of the cubic structure of Fe_2_O_3_, indicating partial phase coexistence. FFT analysis further confirmed the face-centered cubic structure of Fe_3_O_4,_ and the NPs remained single-crystalline, as illustrated in Figure 8b,f by characteristic NPs showing their atomic structure projected along the <011> and <031> zone axes respectively. HRTEM images of the enriched SiO_2_ shell with Ca^2+^ ions [Figure 8d–f] (samples Fe_3_O_4_/mSi/Ca_1A, Fe_3_O_4_/mSi/Ca_1B, Fe_3_O_4_/mSi/Ca_1C) revealed the existence of a Ca/SiO_2_ shell with a slightly increased thickness (~11–20 nm) [Figure 8d,f] and the coexistence of the Fe_3_O_4_–Fe_2_O_3_ phases. STEM-EDX microanalysis was employed to determine the chemical composition of the NPs. The elemental mapping [Figure 9a,b] confirms the presence of iron (Fe, red), oxygen (O, green), and silica (Si, purple) in all samples, while calcium (Ca, blue) is also detected, although in very small amounts. The corresponding EDX spectra and atomic percentage tables further verify Ca incorporation in the Fe_3_O_4_/mSi/Ca samples of procedure 1, supporting the successful introduction of Ca into the mesoporous silica shell, even at low concentrations.

In contrast, synthesis procedure 2 (Fe_3_O_4_/mSi_2 and Fe_3_O_4_/mSi/Ca_2 samples) resulted in a very thin and irregularly distributed amorphous silica shell around the NPs [Figure 7c,g]. In particular, analysis of the Fe_3_O_4_/mSi/Ca_2 sample revealed an amorphous silica shell enriched with calcium Ca/SiO_2_ ions of ~3 nm. HRTEM and SAED analyses, in agreement with all the previously coated samples, confirmed the FCC structure of Fe_3_O_4_ and the cubic structure of Fe_2_O_3_ [Figure 8c,g]. Particularly, the HRTEM image of Figure 8c and the corresponding FFT, given as an inset, of a spherical NP clearly reveal the cubic Fe_3_O_4_ structure projected along the <111> zone axis. Complementary STEM-EDX analysis [Figure 9c] further supported these findings. The EDX spectrum clearly displayed the Fe, O, and Si peaks, followed by a relatively faint Ca peak throughout the particles’ distribution. STEM-EDX mapping indicated that Fe (green) and O (blue) elements were confined to the particles while silicon Si and Ca elements overlapped the aggregated imaged field, confirming that the NPs are coated by a shell around their periphery. The presence of carbon (C) and copper (Cu) peaks was also observed, attributed to a carbon-coated Cu TEM grid.

According to synthesis procedure 2, it seems that the formation of a silica shell enriched with Ca ions around individual magnetite MNPs does not appear to have been successfully achieved, as compared to the first synthesis. Unlike the first synthesis, only a thin, barely visible amorphous layer was observed. While the Fe_3_O_4_ MNPs maintained their crystallinity and structural integrity, significant aggregation of the magnetic particles occurred. This aggregation explains why the mSiO_2_ coating of each nanoparticle was not obtained; instead, the silica shell formed around both aggregated and isolated particles. In general, magnetite and maghemite crystallize in the cubic spinel structure with very similar lattice constants *α* = 8.397 Å and *α* = 8.3515 Å, respectively. Therefore, it is quite challenging to distinguish these two phases.

#### 3.5.2. Size Distribution

**Figure 10:**
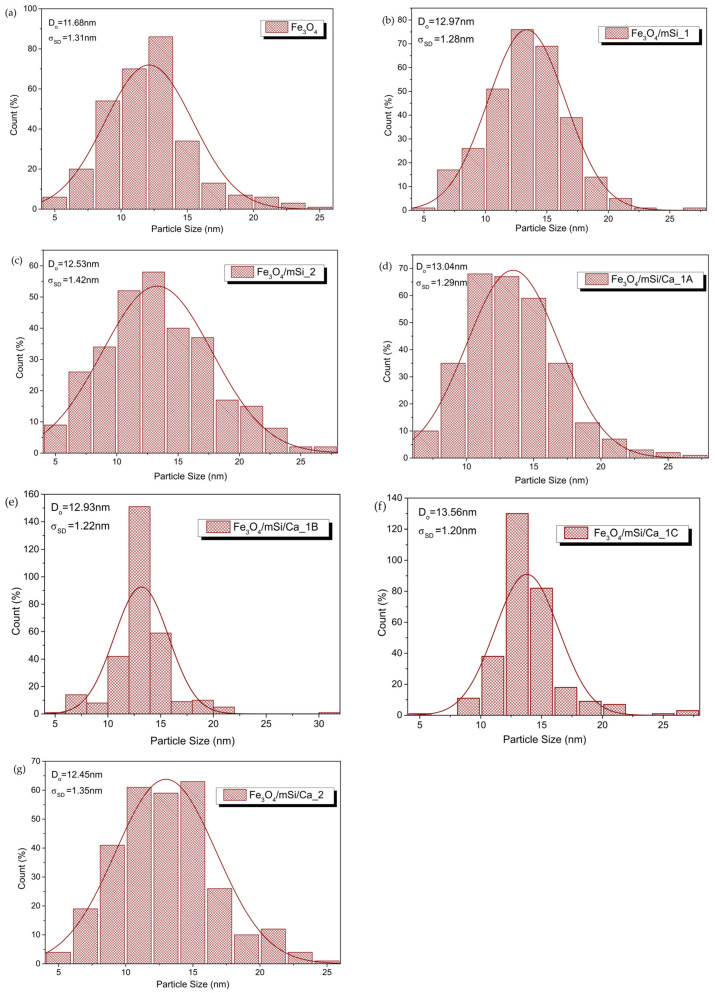
Size distribution, determination of mean size and standard deviation for (**a**) Fe_3_O_4_, (**b**) Fe_3_O_4_/mSi_1, (**c**) Fe_3_O_4_/mSi_2, (**d**) Fe_3_O_4_/mSi/Ca_1A, (**e**) Fe_3_O_4_/mSi/Ca_1B, (**f**) Fe_3_O_4_/mSi/Ca_1C and (**g**) Fe_3_O_4_/mSi/Ca_2 NPs, with log-normal distribution function.

Figure 10 shows the size distribution histograms of MNPs. For a qualitative estimate of their size, the calculation was focused on the magnetic particle cores. The results confirmed the good preservation of the core dimensions (~13 nm).

### 3.6. BET Analysis

**Figure 11:**
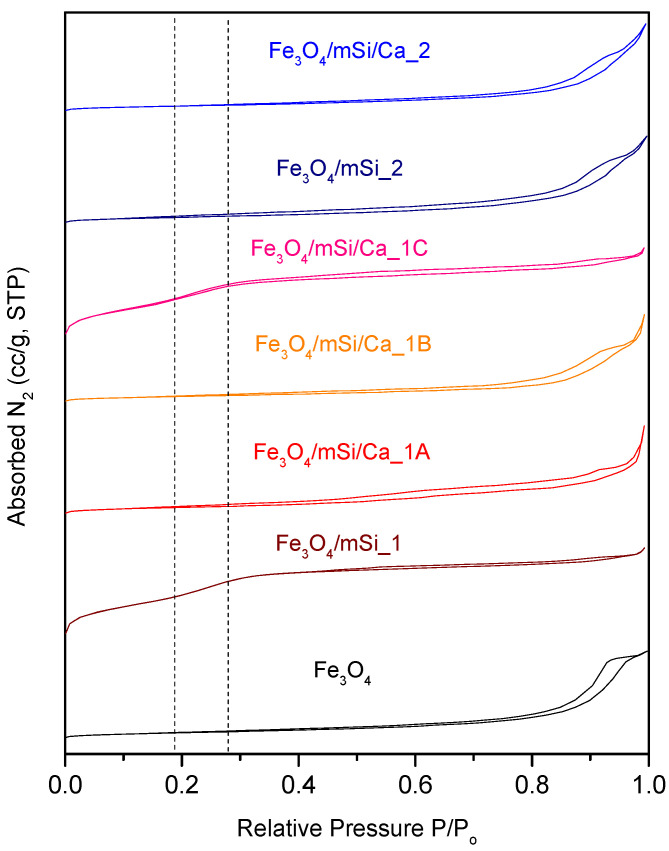
Nitrogen adsorption isotherms of all synthesized NPs.

In the porosity analysis, the N_2_ adsorption step of all samples exhibited a porous-like behavior, yet with significant differences that lead to the assumption of an inter-porosity nature of the synthesized NPs (Figure 11). Fe_3_O_4_ NPs did not present a hysteresis loop, had a range of pore diameters between 4 and 50 nm, while the peak of the curve in BJH analysis has a maximum at 25 nm; both low surface area and pores’ volume were low enough to assume that, unlike a classic type IV(b) isotherm of a MCM-41 material, Fe_3_O_4_ does not contain uniform pores in terms of size neither contain mesoporous structure. After coating with silica shell, the type IV(b) isotherm is formed for the Fe_3_O_4_/mSi_1 but not for the Fe_3_O_4_/mSi_2 sample. For the Ca^2+^ enriched samples, only the Fe_3_O_4_/mSi/Ca created a type IV(b) mesoporous isotherm, with comparable peaks to the previously synthesized Fe_3_O_4_/mSi_1 sample. Fe_3_O_4_/mSi_2, Fe_3_O_4_/mSi/Ca_1A, Fe_3_O_4_/mSi/Ca_1B and Fe_3_O_4_/mSi/Ca_2 samples did not demonstrate a mesoporous structure. However, in the case of the Fe_3_O_4_/mSi_1 and Fe_3_O_4_/mSi/Ca_1C samples, the desired mesoporous shell was developed. All data are presented in Table 4.

### 3.7. Cytotoxicity

**Figure 12:**
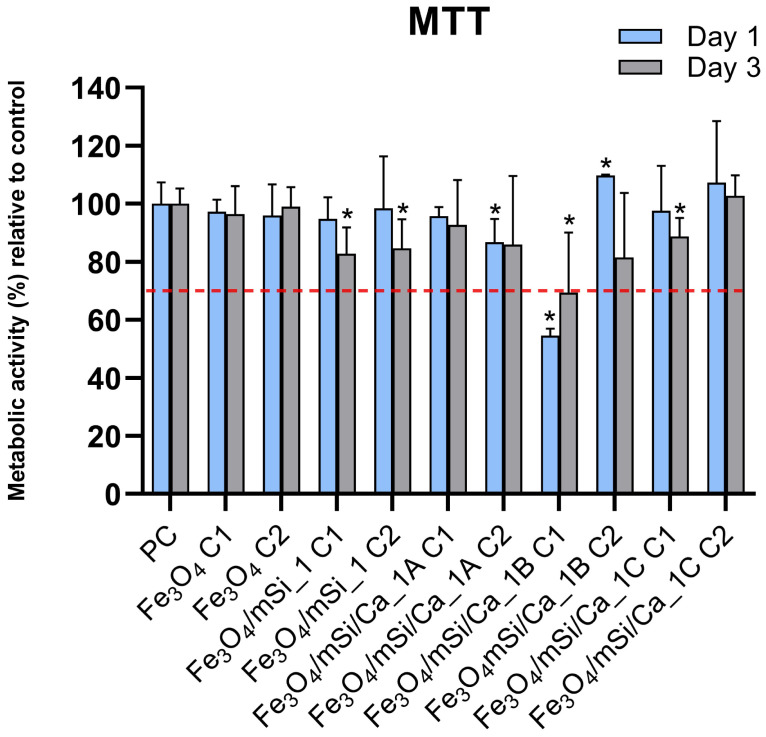
Cytotoxicity test results of the tested MNPs eluate at two different concentrations (C1: 0.5 mg/mL, C2: 0.25 mg/mL).* = statistically significant (*p* < 0.05) compared to PC. The dashed red line indicates the cytotoxicity acceptance threshold defined in ISO 10993-5, corresponding to 70% relative cell viability.

The previously discussed results indicate that the synthesis procedure 1 was more effective in producing an amorphous porous silica shell around MNPs; therefore, cytotoxicity tests were carried out for the core (Fe_3_O_4_), Fe_3_O_4_/mSi_1, Fe_3_O_4_/mSi/Ca_1A, Fe_3_O_4_/mSi/Ca_1B, and Fe_3_O_4_/mSi/Ca_1C samples. Figure 12 presents the % metabolic activity of the cells compared to the control after culture of NPs eluates with HGFs. All samples exhibited similar biocompatibility comparable to control, with the exception of the Fe_3_O_4_/mSi/Ca_1A sample, which showed mild cytotoxicity at the highest tested concentration. Notably, no dose-dependent toxicity was observed in any of the samples, indicating that the biocompatibility of these NPs is not significantly influenced by their concentration within the tested range. The mild toxicity observed in the Fe_3_O_4_/mSi/Ca_1B NPs at higher concentrations highlights the need for further optimization of surface modifications to balance functionality with biocompatibility.

### 3.8. ROS Generation Evaluation

**Figure 13:**
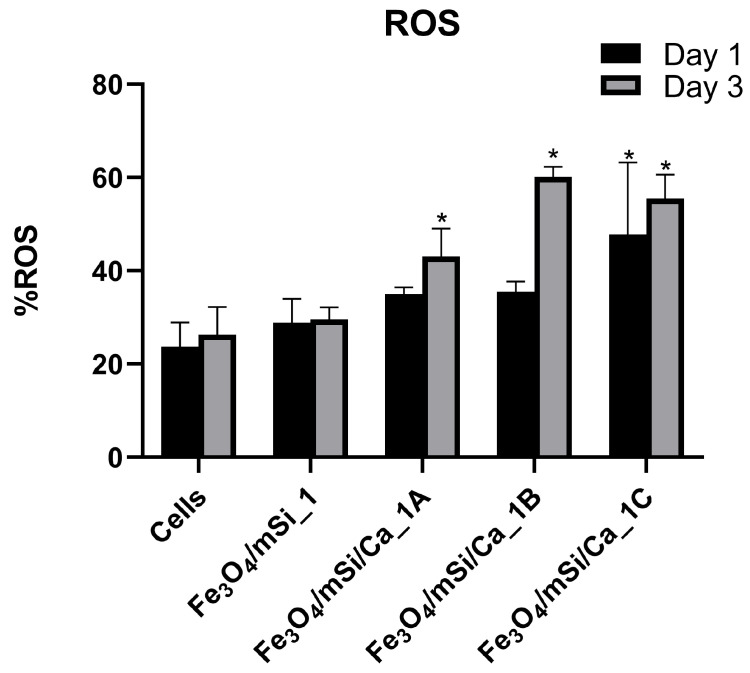
ROS levels of the tested MNPs at concentration C2: 0.25 mg/mL. * = statistically significant (*p* < 0.05) compared to PC.

ROS levels were measured on days 1 and 3 for all samples. The Fe_3_O_4_/mSi_1 group showed only a slight, non-significant increase at both time points compared to the control (Figure 13). In the Ca-enriched samples, ROS values were higher. The Fe_3_O_4_/mSi/Ca_1A sample showed a significant increase on day 3, while the Fe_3_O_4_/mSi/Ca_1B and Fe_3_O_4_/mSi/Ca_1C samples presented the highest ROS levels, with statistically significant differences observed on both days compared to control cells (*p* < 0.05).

### 3.9. Osteogenic Differentiation

**Figure 14:**
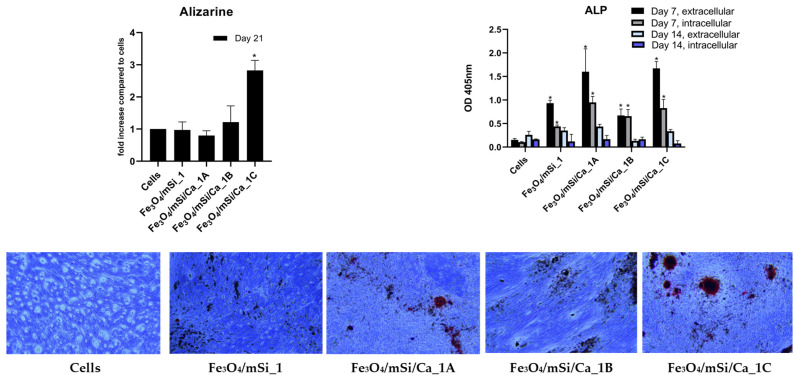
ARS, ALP, and representative images after ARS staining of the tested MNPs. * = statistically significant (*p* < 0.05) compared to cells.

Extracellular and intracellular ALP activity was evaluated on days 7 and 14 (Figure 14). On day 7, all Ca-enriched MNPs exhibited significantly higher ALP activity compared to the cells alone, with Fe_3_O_4_/mSi/Ca_1B and Fe_3_O_4_/mSi/Ca_1A showing the highest extracellular values. By day 14, ALP levels decreased across all groups. The Fe_3_O_4_/mSi_1 sample showed only small changes in ALP activity at both time points. Alizarin Red staining was quantified on day 21 to evaluate mineral deposition. The control cells and the Fe_3_O_4_/mSi_1, Fe_3_O_4_/mSi/Ca_1A, and Fe_3_O_4_/mSi/Ca_1B samples showed similar absorbance values, with no significant differences among them. In contrast, the Fe_3_O_4_/mSi/Ca_1C sample displayed the highest absorbance, with a statistically significant increase compared with the other groups. Fe_3_O_4_/mSi/Ca_1C displayed staining around MNPs, consistent with the quantitative ARS results and confirming its ability to promote mineralization.

## 4. Discussion

Iron oxide magnetic nanoparticles (IONPs) are widely used in the biomedical field due to their low toxicity, ease of synthesis, and surface modification [4,5,16,17]. Magnetite (Fe_3_O_4_), hematite (*α*-Fe_2_O_3_), maghemite (*γ*-Fe_2_O_3_), and wustite (FeO) are classifications of iron oxide. Magnetite is the most widely studied IONP, with a size under 20 nm [26,31], attributed to the reduced relative oxygen concentration. Decreasing NPs’ size leads to a decrease in their ferromagnetic behavior and an increase in their superparamagnetic one. Therefore, Fe_3_O_4_ NPs below 20 nm have distinct superparamagnetic properties and a high surface-to-volume ratio, which is ideal for their use in biomedical applications [28,32].

Among a variety of different synthesis methods, the co-precipitation is a simple and fast preparative method with better control over composition and particle size [29,33]. Iron oxide Fe_3_O_4_ NPs tend to agglomerate and, due to their high chemical activity, oxidize easily, leading to a decrease in their magnetic properties. Indeed, during the experiments, agglomeration occurred, and the observed decrease in magnetization saturation can be attributed to the NPs’ tendency to aggregate, as indicated by TEM.

The coating on Fe_3_O_4_ NPs serves to protect the core by improving stability and preventing oxidation, which can degrade magnetic properties. It also reduces surface energy, minimizing particle agglomeration, and enhances dispersibility. Additionally, the protective shell allows for further functionalization of the nanoparticle surface, enabling versatile applications [31,34]. Mesoporous solid strong bases are easily recoverable and reusable catalysts, offering a wide range of applications and economic viability [14,15,20,22,35]. Moreover, their high surface and abundant porous channels assist adsorbate diffusion and protect active sites from deactivation [21,22,27]. A mesoporous SiO_2_ shell provides additional advantages, such as large pore channels and high pore volume [36,37], while maintaining the core’s magnetic properties in a stable and environment-independent manner. Finally, the shell’s enrichment with Ca ions may induce osteogenesis and promote bone regeneration [27,33,34,35,36,37]. These properties guided the synthesis of Fe_3_O_4_ nanoparticles as magnetic cores, coated with a mesoporous SiO_2_ shell and further enriched with Ca^2+^ ions, for use as drug carriers in bone tissue treatment applications.

### 4.1. Core–Shell Formation

Fe_3_O_4_ was successfully synthesized via the co-precipitation method, and primarily spherical-shaped NPs of an average size of ~12 nm were fabricated. The small mean size of the MNPs (below 20 nm) enhances their potential as superparamagnetic agents for biomedical applications [28,32]. As it comes to MNPs’ coating with a mesoporous silica shell, the two different procedures (modified Stöber and surfactant-templated sol–gel) were attempted to detect which could provide the most optimal properties of the synthesized MNPs.

The synthesis procedure 1 (modified Stöber), indeed, provided a satisfying amorphous silica shell with well-defined thickness on all samples (Fe_3_O_4_/mSi_1, Fe_3_O_4_/mSi/Ca_1A, Fe_3_O_4_/mSi/Ca_1B, and Fe_3_O_4_/mSi/Ca_1C). Coated MNPs were mostly aggregated, however, with good distribution. An oxidation increase after coating was detected as expected, which is translated to the co-existence of the two phases (Fe_3_O_4_–Fe_2_O_3_ [37,38]). In addition, TEM results revealed a multi-core–shell structure of the synthesized MNPs, with only a minor number of magnetic particles individually surrounded/coated by the amorphous shell. A main reason for this is the aggregation of the magnetic particles, which tend to agglomerate during synthesis, due to strong magnetic dipole–dipole attractions, leaving no space for the shell to isolate each particle. If nucleation of silica is slower than core aggregation, the forming shell grows around aggregated cores. Other factors that may contribute to multi-core clustering are the high core concentration in the reaction mixture that increases the likelihood of aggregation before coating and the slow addition of TEOS, which may give time for NPS’ aggregation before the formation of the shell. In the present study, with this synthesis approach, the MNPs were first dispersed in ddH_2_O, and subsequently, CTAB was added, and at a later stage, the TEOS was added drop-by-drop. As magnetic NPs tend to aggregate quickly in water, especially in the absence of a stabilizer, CTAB addition may not be able to surpass this aggregation, fully break the clusters, and isolate each MNP [38,39]. Thus, in the sol–gel synthesis procedure 2, the usage of the organic cosolvent CB was expected to decrease aggregation’s level since CB diffuses into the hydrophobic region of CTAB micelles over time, leading to the structural transformation of bilayer structures. Therefore, a large pore dendritic tail with a more detectable pore size is expected. CB’s diffusion into CTAB micelles is also faster closer to the oil phase (where tails are nucleated) rather than in aqueous solution [37,38,39]. Therefore, the shell’s structure stabilizes better and surrounds the particles more easily while preventing their tendency to aggregate. Truly, better dispersion of the particles was observed, yet the shell was barely defined, without also accomplishing the complete NPs’ isolation and coating by the mSiO_2_, since aggregation still occurred. This might be due to the not strong enough (or not completely built) bonds required for the silica shell’s formation and functionality. In fact, Li et al. [15] also synthesized similar core–shell nanoparticles that indeed presented aggregation yet with a higher magnetic core–particle isolation via their amorphous shell coating. The better-defined silica shell of the first synthesis method, and the undefined shell thickness detected in the 2nd method, are in agreement with the synthesis made before by Zhao et al. and Li et al. [15,16,17].

### 4.2. Characterization Results

XRD analysis revealed that all samples exhibited the characteristic crystal structure of Fe_3_O_4_, with a high degree of crystallinity, which was only slightly decreased after coating the magnetic core with a SiO_2_ shell and further enrichment of the shell with Ca^2+^ in all samples, in agreement with previous experiments [14,15,16,17]. This indicates the good preservation of the magnetic core and its crystal state, yet with a partial conversion of Fe_3_O_4_ to Fe_2_O_3_, which is expected due to MNPs’ ease of oxidation [37,38]. The only new peak in the Fe_3_O_4_/mSi/Ca_1A sample at angles 2θ = 29.35°, along with the weaker ones at 39.35°, 47.5°, and 48.4°, are attributed to calcium carbonate’s formation (calcite, Ca(CO_3_), Jade tab: #860174), indicating excess of calcium not incorporated into the silicate lattice [29,31]. This peak’s existence may be attributed to the higher amount of calcium ions in this sample, with a TEOS:Ca^2+^ ratio of 60:40, since the Fe_3_O_4_/mSi/Ca_1B sample (90:10 TEOS:Ca^2+^) revealed no such diffraction peaks. The higher Ca^2+^ loading may exceed silanol site availability, favoring precipitation of CaCO_3_ during hydrolysis and condensation [40,41]. FTIR analysis verified the presence of silicate peaks in the range of 750–1100 cm^−1^, assigned to the silica coating of the MNPs. The coexistence of the two SPION phases (Fe_3_O_4_ and Fe_2_O_3_), especially in the synthesis procedure 1 (stretching vibration Fe–O at ~450 cm^−1^), comes in agreement with the work of Zinan Zhao et al. [16], where the same two bands at ~440 cm^−1^ and 562 cm^−1^ were attributed to the co-existence of magnetite and maghemite. This was also proven before by Antarnusa G. et al. while synthesizing Fe_3_O_4_ MNPs [17]. In addition to these two studies, Alterary et al. [42,43] synthesized and characterized magnetic core–shell NPs of Fe_3_O_4_ coated with a SiO_2_ shell, and FTIR analysis once again revealed the second band at ~440 cm^−1^. It is then well assumed that the presence of these two SPION phases is nearly unavoidable.

Another interesting observation is that in experimental procedure 2 (Fe_3_O_4_/mSi_2 and Fe_3_O_4_/mSi/Ca_2), the presence of a silicate peak appeared with much lower intensity. Two factors that may have contributed to that are as follows [41,44]: a. the lower water/TEOS ratio in synthesis 1, may have slowed down TEOS hydrolysis, resulting in a thinner shell after condensation; and b. the higher concentration of magnetic NPs in the synthesis II, which may have resulted in lower available silica due to silica distribution to more cores.

### 4.3. Magnetic Properties

VSM measurements of all samples revealed an M_S_ value in the range 7–22 of Am^2^/kg, which lies within the appropriate values for biomedical applications [45,46], and are consistent with reported values for Fe_3_O_4_/mSi magnetic core–shell nanoparticles in previous studies. For example, M_S_ for Li X. et al. and Li T. et al. [14,15] reported values of 34.9, 27.8, and 22.5 Am^2^/kg for Fe_3_O_4_, Fe_3_O_4_/Si and Fe_3_O_4_/Si/Ca NPs, respectively. In another study, Ding et al. [41] found that M_S_ values of silica-coated Fe_3_O_4_ NPs with different shell thicknesses were 30.0, 5.2, and even 1.9 Am^2^/kg. The lower M_S_ on the coated samples is expected because the M_S_ value is found to be proportional to the amount of weight for the same magnetic material [43]. The difference is due to the surface of the samples bearing an irregular magnetic spin due to interactions between Fe–O bonds and incomplete coordination between surface atoms. As for the low magnetization value of the synthesized NPs, it may be due to the oxidation of magnetite to maghemite, a phase of SPIONS with lower magnetization compared to Fe_3_O_4_, as well as disordered spins at the particle surface [17]. The satisfactory magnetization range indicates the material’s ability to separate immediately after acting in aqueous solution [15,16,17].

Moreover, in this work, all samples’ hysteresis loops are so narrow that they make coercivity and remanence difficult to determine. Consequently, both core and core–shell compositions are classified as soft materials, due to the narrow hysteresis curve they form and exhibit ideal soft magnetization at ambient temperature [15,19]. The superparamagnetic behavior of the synthesized NPs can be seen based on the morphology and shape of the hysteresis loops [17,19]. Loop’s curves and shape suggest that small NPs demonstrate high magnetic response, since the small particle of the particles’ grains leads to instability in the magnetic moment of the MNPs, which is due to the small anisotropy energy characteristic of MNPs. Thus, in the case of the application of an external magnetic field, the magnetic moment in small NPs leads to a faster response. In general, an increase in NPs’ size, with any non-magnetic additions (silica shell, calcium ions and so on), implies a decrease in their magnetization. In fact, there is a critical point (threshold) at which NPs become superparamagnetic. It is therefore expected that by adding a non-magnetic coating and enriching it with an additional element (calcium ions in this case), magnetization decreases as observed in all the samples. Moreover, experimental procedure 1 successfully resulted in the formation of an amorphous, well-defined silica shell, of higher thickness in contrast to experimental procedure 2, where the shell was thin and almost undetectable, hence MNPs of samples Fe_3_O_4_/mSi_2 and Fe_3_O_4_/mSi/Ca_2 mainly contained magnetic core particles, which explains the smaller decrease in M_S_. However, experimental procedure 1, despite the higher a % of decrease in M_S_, remained within the required range for biomedical applications. The higher magnetization saturation (M_S_) observed in Fe_3_O_4_/mSi/Ca_1A, compared to the expected decrease of M_S_ in the Fe_3_O_4_/mSi/Ca_1B sample, may be due to a different rate of Ca incorporation into the silica shell. This could be related to the different TEOS:Ca ratios used during synthesis (60:40 for Fe_3_O_4_/mSi/Ca_1A vs. 90:10 for Fe_3_O_4_/mSi/Ca_1B), with the higher Ca content in Fe_3_O_4_/mSi/Ca_1A potentially contributing to a greater reduction in M_S_.

### 4.4. Mesoporous Structure

Type IV(b) is a reversible isotherm of materials with smaller mesopores. These isotherms are created due to conical and cylindrical mesopores closed at the tapered end [27,28]. Considering BET results, all coated samples revealed a poorly developed porous structure, with primarily interparticle porosity, except for the Fe_3_O_4_/mSi_1 and Fe_3_O_4_/mSi/Ca_1C samples. Regarding the Ca-enriched samples, Fe_3_O_4_/mSi/Ca_1A a and Fe_3_O_4_/mSi/Ca_1B, there is a possibility that Ca^2+^ ions are blocking the existing pores and CTAB cannot completely be removed. In the case of Fe_3_O_4_/mSi_1 and Fe_3_O_4_/mSi/Ca_1C, a distinct inflection point (“knee”) appears successfully on the respective isotherms, indicating the existence of a mesoporous type IV(b) amorphous mSiO_2_ shell, enriched with Ca in these two cases. This assumption agrees with the study of Pouroutzidou et al. [27,28], who synthesized mesoporous Mg and Sr-Doped NPs for moxifloxacin drug delivery and calcinated the samples over 550 °C [27,28].

All samples were confirmed to be porous materials. The observed variations in the isotherms reflect differences in particle aggregation, which is more realistic since isolated nanoparticles are difficult to detect due to the formation of multi-core–shell structures. Despite that the first experimental procedure led to the successful synthesis of a mesoporous type IV(b) silica shell (modified Stober method: Fe_3_O_4_/mSi_1), neither the Fe_3_O_4_/mSi/Ca_1A nor Fe_3_O_4_/mSi/Ca_B maintained this structure due to Ca^2+^ ions blocking the existing pores. Thus, the wet impregnation technique (Fe_3_O_4_/mSi/Ca_1C) was employed and indeed formed the desired mesoporous Ca-doped shell. Εxperimental procedure 2 did not result in a mesoporous shell, as shown by its faint presence around the particles, as mentioned before.

Lastly, among all samples, Fe_3_O_4_/mSi/Ca_1C exhibited the highest specific surface area (463.3 m^2^/g), which is comparable to or superior to values reported in similar mesoporous drug delivery systems. For instance, triple-porous Fe_3_O_4_/SiO_2_ microspheres have been reported to reach surface area of ~426 m^2^/g [44], while the Fe_3_O_4_/DPU/MSH hybrid structure achieved surface area of ~386 m^2^/g by Moorthy [46]. Given that surface area directly influences drug loading capacity, this elevated value highlights the superior potential of Fe_3_O_4_/mSi/Ca_1C as a promising candidate for further pharmaceutical applications.

As it comes to size distribution, all samples maintained a mean particle size of ~13 nm, which is acceptable for utilization in biomedical applications. However, since the synthesis approaches resulted in multi-core–shell structures, the measurements were taken for the Fe_3_O_4_ particles embedded within a major amorphous, porous, silica shell. As a result, it was practically impossible to determine the size distribution of isolated core–shell NPs, except in very limited areas, where isolated magnetite cores were fully encapsulated by the shell, as revealed by TEM imaging. This overall coating effect was also detected by Alterary S. et al. [42] in their TEM results, where they synthesized and characterized Fe_3_O_4_-SiO_2_ core–shell nanostructures. Their samples exhibited mainly spherical shapes, yet were also aggregated with a SiO_2_ shell covering larger regions containing coexisting aggregated magnetic cores.

### 4.5. Cytotoxicity—Osteogenic Potential and ROS Analysis

The lack of toxicity in HGFs was verified through indirect toxicity testing. Indirect tests mainly assess toxic effects caused by soluble or released components, and lack of toxicity, indicating good chemical stability and low release of harmful species and residual reagents due to the sol–gel synthesis. However, indirect tests do not capture the physical interactions between NPs and cells, thereby potentially hindering a possible harmful contact-dependent toxicity. Lu et al. and Guo et al. [44,47] carried out direct cytotoxicity tests in L929 fibroblasts for Fe_3_O_4_ and Fe_3_O_4_/mSiO_2_, with low particle doses or short incubation times, and showed little reduction in cell viability, while fibroblast cell viability decreased with the increase in time and concentration. Dose-dependent changes in cell morphology, viability, and apoptosis rate have been observed in other studies, as well as ROS production, challenging the protective effect of even the silica coating [48,49]. Inconclusive results also exist in terms of NPs size [50,51,52,53], highlighting the need for more thorough evaluation of cell toxicity and possible immunomodulating effects.

We selected hPDLCs to evaluate the capacity of our materials to induce osteogenic differentiation. These cells possess osteogenic potential and can readily shift toward an osteoblast-like phenotype when exposed to appropriate biochemical or biomaterial-derived stimuli [24,54]. In the present study, it was demonstrated that the Fe_3_O_4_/mSi/Ca_1C MNPs were more efficient in promoting the hPDLCs osteogenic differentiation in vitro according to higher ALP activity and increased matrix mineralization (ARS). This is probably attributed to the presence of calcium in the silica shell, as verified by XPS. ALP was increased in all samples on day 7 and decreased after 14 days of culture, a finding that was expected, as ALP is an early osteogenic marker. Although mesoporous silica-coated magnetic (Fe_3_O_4_) nanoparticles present remarkable capability in promoting the osteogenic differentiation of mesenchymal stem cells via the Wnt/β-catenin pathway in vitro [55], the long-term mineralization data (ARS, day 21) reveal differences among the MNPs. While Fe_3_O_4_/mSi_1, Fe_3_O_4_/mSi/Ca_1A, and Fe_3_O_4_/mSi/Ca_1B showed ARS values comparable to the control cells, Fe_3_O_4_/mSi/Ca_1C exhibited a significantly higher absorbance, indicating higher calcium deposition. Taken together with the viability (MTT) data, showing overall acceptable cytocompatibility when testing the MNPs eluates, these results suggest that a certain level of ROS generation by the direct contact of cells with the Ca-doped Fe_3_O_4_/mSi nanoparticles does not abolish, and may even accompany, osteogenic differentiation, provided that oxidative stress remains below a cytotoxic threshold. On the other hand, mesoporous silica-coated MNPs can promote osteogenic differentiation through a combination of bioactive ion release, favorable surface properties, and magnetic responsiveness [56]. The gradual dissolution of the silica shell releases soluble silica species known for their capacity to activate osteogenic pathways [50,53] and upregulate markers such as RUNX2, ALP, and osteocalcin, while the high surface area of the mesoporous structure enhances protein adsorption and integrin-mediated cell adhesion, further stimulating osteogenic signaling [55]. The magnetic Fe_3_O_4_ core can also contribute to mechanotransductive stimulation, which has been shown to enhance osteoblast differentiation under static or dynamic magnetic cues [53]. Together, these features create a bioactive microenvironment that supports osteogenic commitment and mineralization of progenitor cells. In our study, the Ca-doped mesoporous silica-coated magnetic nanoparticles similarly induced elevated ROS levels in direct-contact culture, especially in Ca_1B and Ca_1C, yet still Fe_3_O_4_/mSi/Ca_1C supported osteogenic differentiation. This finding supports the concept that moderate ROS levels can be pro-osteogenic, particularly when accompanied by Ca^2+^ and silicate ion release, which further enhances osteogenic gene expression and mineralization. However, further research is needed to capture ROS levels at different stages of differentiation, with thorough monitoring of ion species release to fully elaborate on their role in bone tissue engineering.

### 4.6. Comparative Analysis and Selection of the Optimum Synthesis Method

In this study, we synthesized core–shell magnetic nanoparticles enriched with calcium. Although core–shell structures were achieved in all cases, only two samples exhibited a clear mesoporous silica shell (Fe_3_O_4_/mSi_1 and Fe_3_O_4_/mSi/Ca_1C), while the others showed an interparticle porosity. Different synthesis approaches were employed to collectively combine the desired functionalities. The optimized samples Fe_3_O_4_/mSi_1 and Fe_3_O_4_/mSi/Ca_1C presented mainly spherical shape and size below 20 nm, verifying their suitability for biomedical applications. These samples retained primarily multi-core single-shell structure due to magnetic particle aggregation, retained the crystallinity of Fe_3_O_4_, and although partial oxidation of Fe_3_O_4_ into Fe_2_O_3_ may have slightly reduced magnetization, it remained within acceptable limits. Considering Ca^2+^ addition, the wet impregnation method for calcium enrichment yielded a mesoporous, amorphous silica shell with a uniform thin thickness coating of the magnetic cores. This process also maintained the biocompatibility of the MNPs and preserved the mesoporous type IV(b) structure of the silica shell after Ca-doping, supporting their ability to promote osteogenic differentiation. Among the tested formulations, Fe_3_O_4_/mSi/Ca_1C appears to achieve the most favorable balance between ROS induction, cell viability, early ALP upregulation, and late-stage mineralization, highlighting it as the most promising candidate for bone-regenerative applications. Thus, the aforementioned samples were selected as ideal candidates for further pharmaceutical or biomedical applications.

Achieving good dispersion to form a classic single core–shell structure while maintaining magnetic properties remains a challenge that requires further investigation. To prevent particle aggregation and enhance the stabilization and functionalization of type IV porous shells, research should focus on testing different water/TEOS, NPs/water, and TEOS:Ca^2+^ ratios, as well as calcination times and TEOS addition to the solution. It has been reported that for the same amount of TEOS a slow incorporation (stepwise or on different increments) and a low concentration of magnetic NPs can enhance dispersion and homogeneous coating of isolated NPs [57]. Additionally, the wet impregnation method for calcium enrichment is preferable to ensure the formation and preservation of the mesoporous shell. These approaches should allow micelles to better stabilize the mesoporous structure of the shell compared to current attempts, facilitating drug encapsulation within the pores and avoiding pore blockage by Ca^2+^ ions. Future research should focus on testing biocompatibility with different cell lines, the exploration of their bioactivity in terms of in vitro biological hydroxyapatite formation, their potential immunomodulatory properties, and the encapsulation of pharmaceuticals or biological molecules for controlled release under an external magnetic field.

## 5. Conclusions

Silica-coated, calcium-doped multi-core–shell magnetic nanoparticles were successfully synthesized using different methods. The optimized samples featured a mesoporous amorphous silica shell formed by a modified Stöber method, followed by successful calcium addition via wet impregnation. The other samples exhibited an amorphous, Ca-doped silica shell characterized primarily by interparticle porosity. The coating preserved the core’s crystallinity and magnetic properties but caused partial conversion of magnetite to maghemite, more evident in experimental procedure 1. Procedure 2 (sol–gel) resulted in reduced particle aggregation, but in thinner, less defined shells. Particle size remained around 13 nm throughout the different synthesis routes. In general, the materials were biocompatible, with minor exceptions; a slight toxicity was recorded at the highest concentration of the Fe_3_O_4_/mSi/Ca_1B sample. Their capacity to promote the osteogenic differentiation of hPDLCs further demonstrates their supportive roles in tissue engineering and biomedical applications. Future research should focus on improving particle dispersion, maintaining magnetic properties, and exploring biological functions like hydroxyapatite formation and their capacity for osteogenic differentiation and controlled drug release under static or dynamic magnetic fields.

## Figures and Tables

**Table 1:** Samples names with their chemical compositions.

Samples	Composition
Fe_3_O_4_	magnetic core FeCl_3_•6H_2_O 98%, FeSO_4_•7H_2_O 98%, NH_4_OH 25%
Fe_3_O_4_/mSi_1	Silica shell TEOS:Ca^2+^ → 100:0 TEOS, CTAB, NH_3_ 25 wt%, CH_3_CH_2_OH
Fe_3_O_4_/mSi/Ca_1A	Silica shell Ca-enriched TEOS:Ca^2+^ → 60:40 TEOS, CTAB, NH_3_ 25 wt%, CH_3_CH_2_OH, Ca(NO_3_)_2_•4H_2_O
Fe_3_O_4_/mSi/Ca_1B	Silica shell Ca-enriched TEOS:Ca^2+^ → 90:10 TEOS, CTAB, NH_3_ 25 wt%, CH_3_CH_2_OH, Ca(NO_3_)_2_•4H2O
Fe_3_O_4_/mSi/Ca_1C	Silica shell Ca-enriched (wet impregnation) TEOS:Ca^2+^ → 60:40 TEOS, CTAB, NH_3_ 25 wt%, CH_3_CH_2_OH, Ca(NO_3_)_2_•4H_2_O
Fe_3_O_4_/mSi_2	Silica shell TEOS:Ca^2+^ → 100:0 TEOS, CTAB, C_6_H_15_NO_3_, C_6_H_5_Cl
Fe_3_O_4_/mSi/Ca_2	Silica shell Ca-enriched TEOS:Ca^2+^ → 60:40 TEOS, CTAB, C_6_H_15_NO_3_, C_6_H_5_Cl, Ca(NO_3_)_2_•4H_2_O

**Table 2:** Quantification results of Fe_3_O_4_/mSi_1 and Fe_3_O_4_/mSi/Ca_1C MNPs.

**Fe_3_O_4_/mSi_1**									
State #0	Etch Time	0.00 s							
Peak		Type	Position	FWHM	Raw Area	RSF	Atomic	Atomic	Mass
			BE (eV)	(eV)	(cps eV)		Mass	Conc %	Conc %
C 1s		Reg	284.720	2.001	2867.1	0.278	12.011	6.51	4.33
O 1s		Reg	533.120	2.032	100,069.5	0.780	15.999	75.05	66.55
Fe 2p		Reg	711.070	0.274	1377.6	2.957	55.846	0.26	0.80
Si 2p		Reg	103.920	1.870	9052.6	0.328	28.086	18.19	28.32
Ca 2p		Reg	352.670	0.000	0.0	1.833	40.078	0.00	0.00
**Fe_3_O_4_/mSi/Ca_1C**									
State #0	Etch Time	0.00 s							
Peak		Type	Position	FWHM	Raw Area	RSF	Atomic	Atomic	Mass
			BE (eV)	(eV)	(cps eV)		Mass	Conc %	Conc %
C 1s		Reg	284.770	2.741	8620.8	0.278	12.011	22.62	15.22
O 1s		Reg	532.870	2.307	67,094.5	0.780	15.999	58.18	52.17
Fe 2p		Reg	711.220	1.377	2813.8	2.957	55.846	0.61	1.90
Si 2p		Reg	103.470	1.902	7088.3	0.328	28.086	16.47	25.92
Ca 2p		Reg	347.220	2.190	5412.2	1.833	40.078	2.13	4.78

**Table 3:** Saturation magnetization values for each synthesized sample of core–shell MNPs.

Sample	M_S_ [Am^2^/kg]
Fe_3_O_4_	63.0
Fe_3_O_4_/mSi_1	39.5
Fe_3_O_4_/mSi/Ca_1A	60.00
Fe_3_O_4_/mSi/Ca_1B	32.3
Fe_3_O_4_/mSi/Ca_1C	18.7
Fe_3_O_4_/mSi_2	54.1
Fe_3_O_4_/mSi/Ca_2	30.4

**Table 4:** BET/BJH data of all samples.

	Fe_3_O_4_	Fe_3_O_4_/mSi_1	Fe_3_O_4_/mSi/Ca_1A	Fe_3_O_4_/mSi/Ca_1B	Fe_3_O_4_/mSi/Ca_1C	Fe_3_O_4_/mSi_2	Fe_3_O_4_/mSi/Ca_2
Surface Area (m^2^/g)	85	355.4	125.9	49.489	463.3	100.4	82.6
Pore Diameter (nm)	25	2.52	-	16.0–30.1 (absorption)	-	18.7 (absorption)	29.5 (absorption)
				18.7 (desorption)	2.18 (desorption)	19.1 (desorption)	18.2 (desorption)
Total pore volume (cc/g)	0.38	0.409	0.5563	0.278	0.415	0.5042	0.4621

## Data Availability

All data are presented in figures.

## Abbreviations

The following abbreviations are used in this manuscript:

Fe_3_O_4_	Magnetite
Fe_2_O_3_	Maghemite
mSiO_2_	Mesoporous Silica
Ca^2+^	Calcium Ions
NPs	Nanoparticles
MNPs	Magnetic Nanoparticles
MRI	Magnetic Resonance Imaging
Ca_10_(PO_4_)_6_(OH)_2_	Hydroxyapatite
PEG	Polyethylene Glycol
FeCl_3_•6H_2_O	Ferric Chloride Hexahydrate
FeSO_4_•7H_2_O	Ferrous Chloride Tetrahydrate
NH_4_OH	Ammonium Hydroxide
TEOS	Tetraethyl Orthosilicate
CTAB	Cetyltrimethyl Ammonium Bromide
CH_3_CH_2_OH	Ethanol
Ca(NO_3_)_2_•4H_2_O	Calcium Nitrate Tetrahydrate
C_6_H_15_NO_3_	Triethanolamine
C_6_H_5_Cl or CB	Chlorobenzene
dd-H_2_O	Double-Distilled Water
Ar	Argon Gas
NH_3_	Ammonia
XRD	X-ray Diffraction
FTIR	Fourier-Transform Infrared
KBr	Potassium Bromide
VSM	Vibrating Sample Magnetometer
TEM	Transmission Electron Microscopy
HRTEM	High Resolution Transmission Electron Microscopy
DMEM	Dulbecco’s Minimal Essential Medium
HGFs	Human Gingival Fibroblasts
hPDLCs	Human Periodontal Ligament Cells
DMSO	Dimethyl Sulfoxide
fcc	Face-centered Cubic
BET	Brunauer-Emmett-Teller theory
BJH	Barrett–Joyner–Halenda
XPS	X-ray photoelectron spectroscopy
ARS	Alizarin Red Staining
ALP	Alkaline Phosphatase Activity
ROS	Reactive Oxygen Species
ART	Artemisin
PC	Positive Control
